# Chicago Medical Society

**Published:** 1887-02

**Authors:** 


CHICAGO MEDICAL SRCIETY.

*Stated Meeting, Dec. 6th, 1886.*—THEPRESIDENT, EDMUND J. DOERING, in the chair.

PROFESSOR JOHN A. ROBISON read a paper on the antipyretic action of antipyrin and thallin, which appeared in the January nnmber of the *Journal and Examiner*, and in which he had collected and condensed the results of the experiments of a large number of clinicians. The largest number of observers agree that antipyrin is a safe, efficient and unobjectionable antipyretic. Contrary to believe in general it does not have a depressing or debilitating effect on the heart, whereas thallin has. Antipyrin causes no change in the blood, whereas thallin has a deleterious effect on the blood and veins. In conclusion, the author believed antipyrin should always be given to produce apyrexia in cases where the temperature is excessively high. He does not believe the drug has any power to lessen or prolong the duration of the continued fevers. He also stated that it not only lowers the temperature, but relieves the pain that accompanies acute articular rheumatism with endocarditis, especially if given with alkalies.

DR. J. J. M. ANGEAR said he had not given this subject any special study, but the subject of temperature he had, and it might not be amiss to speak of some difficulties against which we have to contend. If radiation is increased or diminished and metabolism remains normal, we have increased or decreased temperature; or if metabolism of the body be increased or diminished and radiation remains normal, then we have increased or decreased temperature. We have no means of arriving at any definite conclusion whether our patient, with increased temperature, is suffering with increased metabolism or decreased radiation, or both. We know from observation and from various experiments that the internal part of the body is very frequently hotter than normal, during the cold stage of fever, and cooler than normal during the febrile stage. May not the sum total of caloric in the body be diminished when the thermometer tells us that it is increased, and *vice versa?* If we answer yes, then we are found trying to diminish the temperature, when we should be husbanding what we have. Does not this show that there is a certain amount of pathological knowledge in regard to body temperature at which we have not yet arrived? And our therapeutical knowledge is necessarily as defective in this regard.

Do antipyretics facilitate radiation or check metabolism, or both, or do we know anything about it except its action upon the thermometer? If we could arrive at some definite, pathological conclusion as regards the pyrexial state of the patient, then we could, perhaps, arrive at some scientific knowledge of the action and use of all antipyretics. Had we an instrument by which to measure the amount of radiation and metabolism, it would be of far greater value to us than the thermometer.

PROFESSOR G. C. PAOLI said: The paper was well prepared and interesting, but the question arises, do we know anything about the essential nature of fevers? He would answer, no. But we know the effect, if we do not know the nature, and infevers we generallly use remedies to lessen the temperature. He had used antipyrin in only eight cases: two of them typhoid fever, two of scarlatina, two of lung trouble. In regard to the fever, he admitted that the remedy had an antipyretic effect. In two cases the patients vomited as an effect of taking this remedy. He commenced with ten grains, and it diminished the temperature but did not stop the course of the fever, which lasts usually from five to six weeks. One effect he found from the use of antipyrin, is the lessening of nervous irritability. In a case of tuberculosis, after using antipyrin he began with quinine and found it more of a tonic. In the cases of scarlatina in which he used antipyrin he did not observe any favorable effect; it lessened the temperature, of course, but the same effect was produced by quinine.

DR. H. GRADLE said: Although his line of practice does not often enable him to watch the effects of antipyretic drugs in disease, yet he said he could speak of the effect of antipyrin in one disease, viz., the dentition fever of infants; which, although not usually serious, is in a great many instances annoying, and in which, when the fever is high, there is a possible danger of convulsions. He had never heard of any drug that will reduce the fever under these circumstances as promptly and with as little disturbance as antipyrin. With a temperature of 103° F. a child can be rendered quiet in the course of an hour by one dose of four grains, and perhaps a second dose of two grains, and be pacified for the entire night. He desired to ask the author if he has had any experience in the continued use of thallin in typhoid fever, with the effect of aborting the disease? According to some rather startling statements made several months ago by Ehrlich, it was claimed that it reduced the fever permanently in the course of eight to twelve days.

DR. G. W. WEBSTER favored the use of antipyrin. He had used it in cases of typhoid pneumonia. One point is the favorable action of antipyrin on the headaches of typhoid fever. One of the most distressing symptoms when the temperature is very high is the severe, continued headache and the delirium which always accompanies it. He had not found any other remedy that would have as good an effect in these cases as antipyrin, which always relieved the headache promptly.

DR. H. T. PATRICK said that he is quite partial to antipyrin and has used it in a number of cases. It will often effectually reduce the temperature when baths and sponging will not. In a case of pneumonia in a child of eight years he gave antipyrin from the beginning to the end, with good results, except for one day when he tried sponging instead. The child was sponged off six times in six hours, five to ten minutes each time, without any reduction in temperature. Sometimes cold baths have no effect in reducing the temperature because of a very thick panniculus of adipose tissue. Here antipyrin is indicated. The continuous administration of antipyrin in tuberculosis he had found in a few cases very gratifying. In one case ten to fifteen grains were given every afternoon for a term of ten weeks, with great relief to the patient. His temperature was 103° F., and there was prostration and irritability of mind. The antipyrin was a relief to the patient, and to his family on account of the improvement in his disposition.

DR. J. FRANK had had a personal experience with antipyrin, having taken it two years before while suffering with typhoid fever. The temperature ran as high as 106° F., and quinine had no effect in reducing it. He took antipyrin in 15 and 30 grain doses, first by mouth and then by rectum, with the effect of rapidly reducing the temperature. He has used it in practice in the treatment of erysipelas and typhoid fever, but he does not depend upon it entirely, giving it when the temperature rises very high. In erysipelas he has always found that the antipyrin will reduce the temperature.

DR. C. W. LEIGH said he had seen antipyrin used in a limited number of cases, three or four, of typhoid fever, and none of the patients were older than twenty-one. In these cases there was a visible decrease in temperature and the delirium was lessened very much.

PROFESSOR ROBISON said that he had not pretended to give anything except the results of the observations of a large number of physicians who had used this drug and recorded their experiences. Also the results of quite a number of experiments of therapeutists, as for instance Umbach's experiments in regard to the decrease of tissue-waste. He did not try to detail all these different experiments, but attempted to epitomize the well known actions of the drugs that have been observed. Two of these actions, the increase of heat radiation by the dilitation of small blood-vessels, and the decrease of oxidation, are the main factors in producing the fall of temperature. Dr. Angear says if we can arrive at some conclusion in regard to the manner in which the drug acts, we can use it. Professor Robison thinks we have arrived at a conclusion; these experiments have been carefully conducted and we have seen that antipyrin actually reduces high temperature without any danger to the patient, and it is only in cases where the high temperature itself is a danger to the patient that its use in large doses is to be recommended. In regard to its use in aborting typhoid fever, Professor Robison had not tried it except in four cases; one was a boy under seventeen and another one fourteen. The temperature was about 103° F. He gave large doses and in three days all signs of fever had disappeared. A few days afterwards another member of the same family was taken sick, and the antipyrin was used as before, but that patient went through all the stages of typhoid fever. In another family there were five cases of typhoid fever. Two of the family had had the fever, and one of them died; then three others took sick. Antipyrin was given in large doses in all these cases, but it failed to abort the fever in any of them.

DR. HENRY GRADLE read a paper entitled

DISEASES OF THE VAULT OF THE PHARYNX, which appeared in the January number of the *Journal and Examiner.*

PROFESSOR W. E. CASSELBERRY said he agrees with the author on the importance of the pathological states which occur in the naso-pharynx, and would particularly emphasize the importance of the subject in connection with ear diseases. It is usually neglected because the practitioner does not use the rhinoscope. This can readily be used since the introduction of cocaine; formerly, the pharynx being so irritable, it was a different matter, and considerable practice was necessary in order to acquire the use of the mirror, but now a 2 per centum solution of cocaine sprayed around the pharynx will so reduce the sensibility that the introduction of the rhinoscopic mirror is comparatively easy. He was somewhat surprised at the size of the mirror recommended; usually a small one about one-half inch in diameter is used. This, of course, will not show the entire naso-pharynx at one time, but it can be easily rotated from side to side. With a large mirror a hook is always necessary, which usually complicates the case and is more apt to gag the patient than the use of a small mirror without the hook. He is accustomed, in considering the diseases of the naso-pharynx, to divide the inflammatory affections into three classes. First, the simple chronic naso-pharyngitis; second, the hypertrophic naso-pharyngitis, and third, the atrophic naso-pharyngitis. In the first there is a simple inflammation of the mucous membrane and of the connective tissue beneath the membrane, the connective tissue being sometimes involved to such an extent as to form, here and there, thickened areas of infiltration. He thinks that many of the cases in which the author sees no perceptible lesion in the naso-pharynx can be graded in this class. The hypertrophic form of disease may be sub-divided into two classes, one in which the follicular structures are uniformly hypertrophied, the group of follicles constituting the tonsil of Luschka being enlarged and appearing in the rhinoscopic mirror of about the size and configuration of an ordinary tonsil of the throat. And the other sub-class in which the enlargements are pendant, pear-shaped bodies resembling stalactites, and hence called the stalactitic form. This is the form which was originally described under the name of adenoid vegetations, and the one which is met with most commonly in Germany, whilst the uniform enlargement is more common in this country. The atrophic naso-pharyngitis, as is the case in the pharynx itself, may be regarded as a later stage of the hypertrophic naso-pharyngitis, although in rare instances it may commence in the atrophic form. During the hypertrophic stage the proliferated connective tissue cells gradually encroach upon the glandular structures, and, so to speak, squeeze them to death, whilst the connective tissue cells themselves, as in cirrhosis of other organs, of the liver, for instance, later contract and atrophy. The mucous membrane is now pallid and thin, and is encrusted over with a dry secretion. This is the form of disease which so often gives rise to fetor of the breath, especially when it involves the naso-pharynx, although the disease is not restricted to that locality. In reference to the treatment there is little to add to what has already been said. He had not employed the curette much as his patients will not tolerate the blood and pain. The pain now is not as great as before the introduction of cocaine, but the blood horrifies the younger patients, and the parents. He has found an equally efficient, although a more lengthy treatment, in the galvano-cautery. It is bloodless and painless. In cases of stalactitic growths the best method is the galvano-cautery snare. For the uniformly enlarged variety he employs a Hemming naso-pharyngeal electrode. Nitrate of silver as a means in the treatment of catarrhal conditions, although it has been greatly abused, is unquestionably effective if properly used. In cases of simple chronic inflammation of the mucous membrane, where there is thickening of the subcutaneous tissue, a strong solution applied by means of the cotton swab, 40 grains to the ounce, used by the physician every day, then every two days and finally twice weekly, will lead to the absorption of the infiltrated material. Strong solutions of nitrate of silver (40 gr. to ℥i) cause absorption of infiltration, whilst weak solutions (10 gr. to ℥i) stimulate the further production of infiltrated material and the activity of the glands. Consequently weak solutions will only aggravate the hypertrophic form of disease. For the same reason a weak solution will benefit the atrophic form. In reference to the pharyngeal bursa, he expressed the opinion that too great importance has been ascribed to it.

PROFESSOR F. O. STOCKTON said his experience in the treatment of chronic nasal pharyngitis by the use of nitrate of silver had been anything but pleasing, and he had never seen a beneficial result from it. It is recommended by many in the form of powder, 1 grain of nitrate of silver powdered with some drug and distributed over the membrane ; but his experience had been negative. He had found solutions of it to be of no benefit whatever. It discolors everything, is a dirty, useless drug, and greatly overestimated. The use of the curette in removing adenoid vegetations is quite a recent thing. He had not tried the method that the author speaks of, to any great extent, and he prefers the gouge forceps. He generally uses the galvano-cautery, as it is now possible to use it without difficulty with the aid of cocaine, and the fact that there is no blood lost is a very important consideration in the treatment of young subjects, because, even if they have no pain, if they see a drop of blood they are frightened and begin to scream.

DR. M. R. BROWN said in regard to the size of the mirror recommended by the author, he would consider it large, and apt to produce gagging by coming in contact with the pharyngeal walls, but if cocaine is used this may be avoided ; and should a mirror about half the diameter of the one shown be employed in posterior rhinoscopy, nine out of ten instead of three out of four cases can be examined at the first visit.

Referring to the pharyngeal tonsil producing bronchitis and cough, the irritating mucous finding its way into the larynx has a great deal to do with producing laryngitis, which will give rise to cough. The case of bronchitis mentioned by Dr. Gradle was evidently of a reflex nature. After having treated the pharynx with nitrate of silver for any length of time, there results a thickening of its mucous membrane. He believes that nothing is gained from the use of this remedy in the conditions mentioned. Such has been his experience after having employed it in solutions of various strength in the different diseases of the pharynx. He occasionally makes use of the cold-wire snare, but prefers the galvano-cautery. He also employs a gouge or curette with satisfactory results; but,owing to the pain it causes the patients, he has devised a punch forceps, the use of which is attended with less pain than, and is as satisfactory as, the gouge.

Regarding the hypertrophied tonsils receding between the ages of 25 and 30 years, he said he had seen a number of cases in which the patients with this growth were beyond this age. That day he had examined a man 35 years of age, and a short time since removed a similar growth from a man who was over 50 years old.

Speaking of the microscopic appearance of the naso-pharyngeal growths, Mackenzie says that in adenoid vegetations "the glandular element is, as a rule, more marked in growths taken from the vault of the pharynx, whilst in vegetations taken from the lateral walls the stroma of Kis is found in greater abundance."

DR. GRADLE said: As to the size of the mirror, of course where the patient will not permit you to use a large one you will have to use a small one; but where you can use the large mirror, there is an advantage in gaining a full view of everything. In speaking of being able to examine three out of four, he referred to patients as they come. As a rule, he does not use cocaine in the first examination, because it is so disagreeable. he does not use the hook where the distance between the posterior wall and the pharynx is considerable. As regards nitrate of silver, there are many conditions in which it is entirely useless. One is the practice of cauterization with the solid stick. he has never done this. But there are certain conditions where nitrate of silver is useful and acts promptly, viz., chronic hypertrophic catarrh. And it is sure to give relief in the more acute forms of catarrh, where the pharyngeal tonsil is not enlarged enough to cause damage, but swells from temporary congestion. The patient can use a preparation of 4 per centum and apply it himself with a brush, but it is preferable to have the physician use it. The spray, according to his experience, is more effective and not so disagreeable as a dry powder, but it requires considerable care on the part of the physician to avoid staining the clothing.

PROFESSOR F. E. WAXHAM reported a case of

PSEUDO MEMBRANOUS LARYNGITIS, TREATED BY ELECTROLYSIS.

Almost every disease of the human body has been treated by electrolysis, but the larynx has only recently been invaded. It seems probable that stenosis of the larynx from membranous exudation may be reduced by the galvanic current, the false membrane being destroyed or detached. On October 26 the author was called to see a child of 8 years who had been sick nearly a week with membranous croup. She was in the last stages of asphyxia, and all hope of her recovery had been abandoned. An intubation tube was threaded with platimum wire and used as a negative electrode, and introduced into the larynx without difficulty, when respiration at once became easy. The child coughed up considerable soft membrane and ropy mucus and became conscious. The platinum wire was then insulated by passing it through very small wire tubing, and the projecting wire was attached to a twelve-cell McIntosh galvanic battery. A positive electrode which consisted of an ordinary sponge moistened with warm water was placed over the larynx, and a current from ten cells employed. The current caused some pain and considerable redness of the skin under the sponge, and was reduced to eight cells and passed for five minutes, during which time considerable mucus and softened membrane were expelled. The tube was withdrawn, and the child drank two glasses of milk and passed into a quiet sleep. About two hours later, the respiration having become labored, electrolysis was again performed in the same manner, a current from first eight and then ten cells being employed. The effect of the current was to detach and expel patches of false membrane. No further experiments were made with electrolysis. The child died on October 28.

It was expected that the galvanic current would have but little effect upon the false membrane, but that it would relieve the swelling and congestion of the tissues. On the contrary, the current had a decided effect in loosening and detaching the membrane, and the secondary effect was to increase the swelling and congestion.

Experiments were made on two rabbits with a view of ascertaining the effect of the galvanic current on the healthy larynx. One was given chloroform, the tube introduced into the larynx and a current from eleven cells passed for five minutes. Some dyspnœa followed which was attributed rather to rough usage than to the effect of the current. This subsided and the rabbit recovered. The other rabbit was given a current from eight cells, and apparently suffered no inconvenience.

The author thought no deductions could be drawn from a single case; that the current might have been too strong, or not strong enough; that possibly it may be necessary to use an anæsthetic and employ a current strong enough to verge on cauterization. These points could be decided only after further investigation.

PROFESSOR W. E. CASSELBERRY said he considered the report an extremely interesting one, and suggested that the experiments be carried further; to ascertain if the galvanic current has any effect in softening the false membrane outside of the body.

PROFESSOR FRANKLIN H. MARTIN said he had used electrolysis for about three years in the treatment of strictures of the urethra, stenosis of the uterine canal, chronic inflammatory exudations surrounding the uterus, and in fibroid tumors. In listening to this interesting report the question occurred to him whether the effect of loosening the croupous membrane could be attributed to the electrolytic effect of the current of electricity, or merely to the mechanical effect of the electrode. Electrolysis describes the terms upon which it acts; in the case reported we get no evidence of this action. He should be inclined to attribute any beneficial effect that might have occurred to a counter-irritant effect of the positive sponge electrode that was situated externally. In regard to the power of electricity to dissolve substances similar to the exudate found here, he should judge from experience that it possesses that power. He had been told that a current of electricity passed through a culture of bacteria had the effect of destroying the life of the germs. May this not explain an action that might be worth considering in troubles similar to the one under consideration?

DR. J. FRANK suggested that if the author had used a weaker current he would have had a better result. In stricture of the urethra five cells of the McIntosh battery, with the fluid reduced one-half with water, are used. Twelve cells could hardly be borne on the skin. It would dissolve the membrane and produce inflammation.

